# Creating one problem to try and fix another: the saga of ischemic preconditioning

**DOI:** 10.1002/brb3.170

**Published:** 2013-09-15

**Authors:** Louis R Caplan

Please note that an article related to this editorial, “The role of remote ischemic preconditioning in the treatment of atherosclerotic diseases,” doi: 10.1002/brb3.161, can be found here, also published in *Brain and Behavior*.

The best-laid schemes o' mice an' men, Gang aft agley, An' lea'e us nought but grief an' pain for promis'd joy!

–*To a Mouse* by Robert Burns (1785)

In this issue of *Brain and Behavior*, Vasdekis et al. (2013) thoroughly review the theory behind remote ischemic preconditioning and the results to date of its application among patients with atherosclerotic narrowing of arteries supplying various organs including the brain. In these various trials and observations, clinicians and researchers artificially created ischemia to limbs to reduce ischemic injury to organs threatened by preexisting atherosclerotic lesions. By doing so, they, in effect, created one pathological condition (albeit theoretically a completely reversible one) to treat another persistent condition.

I plan in this editorial to place ischemic preconditioning into a historical context, to critique its potential benefits, risks, and limitations, and to try to look ahead at its future applications if any.

## Rationale and Early Studies

The idea behind creating one pathological condition to treat another dates back at least to Hippocrates who prescribed hot water and steam baths to create fever to treat spasticity and pain (Bierman 1942). Wagner-Jauregg received the Nobel Prize for inoculating malarial organisms into individuals diagnosed with syphilitic general paresis (Bierman 1942); Brown-Sequard lit fire to the skin of the trunk to treat spinal cord injuries (McCullough 2011); doctors used bees to sting patients with multiple sclerosis. The unifying concept in creating a new problem was that pathological conditions induced changes in the body that might be effective in treating other preexisting conditions.

The idea behind ischemic preconditioning in the brain arose from two clinical studies that observed that patients who had strokes preceded by transient ischemic attacks (TIAs) in the same vascular territory, often had less severe deficits at the onset of their strokes and more favorable outcomes (Weih et al. 1999; Moncayo et al. 2000). The authors of these studies posited that the preceding ischemia induced adaptive cellular responses to a subsequent further ischemic challenge. An alternate interpretation is that those patients who had preceding TIAs in the same vascular territory had different pathological vascular lesions and pathophysiology than those patients with sudden onset strokes who had no preceding TIAs. Those with preceding TIAs had occlusive in situ vascular lesions. During the interval between the first TIA and the stroke, time might have sufficed to allow good collateral circulation to develop which minimized the size of the infarct that developed when the stenotic artery finally occluded. In contrast, sudden strokes without preceding TIAs were usually embolic and there had been no time for collaterals to develop. So the authors were comparing apples and pears – two dissimilar pathophysiologies – in situ thrombosis versus embolism.

Wegener and colleagues attempted to study the issue of whether collateral circulation explained the favorable outcome in patients with preceding TIAs by analyzing the volume of perfusion-weighted magnetic resonance imaging (MRI) abnormalities shown within 12 h after stroke onset (Wegener et al. 2004). They studied 13 patients with preceding TIAs and compared infarct volumes and perfusion deficits with 41 patients who did not have preceding TIAs. Infarct volumes were smaller in those with preceding TIAs but the size of the restricted perfusion was similar in the two groups. They concluded that improved perfusion was an unlikely explanation for the more favorable outcomes in the preceding TIA group, a conclusion that supported the ischemic preconditioning hypothesis (Wegener et al. 2004). However in this study, the numbers of patients were small and unbalanced (41 vs. 13), there were more patients with internal carotid artery occlusive lesions in the TIA group, and controversy surrounds the ability to quantify the severity of perfusion deficits by perfusion-weighted MRI protocols.

Kirino (2002) reviewed the experimental animal results concerning ischemic preconditioning and offered two potential explanations: (1) a persistent effect on brain neurons – posttranslational modification of proteins or by expression of new proteins via a signal transduction system to the nucleus. These cascades of events may strengthen the influence of survival factors or may inhibit apoptosis and/or (2) a biochemical stress response – the “synthesis of stress proteins may lead to an increased capacity for health maintenance inside the cell. These proteins work as cellular ‘chaperones’ by unfolding misfolded cellular proteins and helping the cell to dispose of unneeded denatured proteins (Kirino 2002).”

It has long been well known that acute phase reactions were generated after many pathological insults. These reactants included many cytokines and other biochemical substances; the increased Factor VIII, and fibrinogen levels increased blood coagulability. Vasdekis and colleagues (2013) in their review identify many potential reactants (opioids, nitric oxide, adenosine, bradykinin, catecholamines, heat shock proteins, heme oxygenase, tumor necrosis factors – α (TNF-α), angiotensin, prostaglandins, hydrogen sulfide, nitrous oxide, and interleukins). This list reads like the usual suspects proffered to explain features of most neurological conditions. Animal studies have shown that these agents are active in ischemic models.

## Ischemic Preconditioning Studies in Humans

The review of Vasdekis et al. contains extensive data about the studies performed to date (Vasdekis et al. 2013). Most often the preconditioning involved causing transient upper or lower limb ischemia shortly before a procedure or surgery. The preconditioning was targeted for an acute short-term effect. The procedures studied were (1) open heart surgery in infants, children, and adults in whom heart, lung, and kidney protection from injury was studied; (2) before coronary artery stenting in which the extent of myocardial damage was monitored; (3) angiography in patients with kidney disease and before renal transplantation – the target organ studied was the kidney; (4) before aortic aneurysm repair – targeting renal, myocardial, and intestinal injuries.

Only two studies involved patients with neck or intracranial stenotic lesions. One sought an acute effect – transient limb ischemia was induced before carotid endarterectomy in order to reduce the frequency and extent of intraoperative hypotension. Only one study had a more chronic and persistent brain protection target and, unlike all of the other studies, involved patients who were known to have brain ischemia. This was a randomized clinical trial in which 68 Chinese patients who recently had a stroke or TIA attributable to intracranial arterial stenosis were studied (Meng et al. 2012). Upper limb ischemic preconditioning was performed among 38 patients. The preconditioning protocol was five cycles of bilateral upper limb ischemia for 5 min followed by reperfusion for another 5 min, performed twice a day for a total of 300 consecutive days. An electronic autocontrol device was used in the preconditioning. The frequency of stroke, TIAs, and cerebral perfusion were compared with 30 patients who had the same inclusion criteria but no preconditioning. The use of antiplatelets, lipid control agents, and antidiabetic drugs was the same in both groups. The incidence of recurrent stroke with positive brain imaging at 90 and 300 days was 5% and 7.9% in those preconditioned and 23.3% and 26.7% in the control group, respectively (*P* = 0.01 each). The frequency of TIAs was also less in the preconditioned group. Brain perfusion was studied using single photon emission computed tomography; 31.6% of preconditioned patients versus 6.7% of the control group had improved perfusion at 90 days (*P* = 0.012), and 76.3% (29/38) of preconditioned patients versus 53.3% (16/30) of controls had improved perfusion at 300 days (*P* = 0.01) (Meng et al. 2012). The published report did not include follow-up studies of the effect of the preconditioning on the limbs used.

## Conclusions

The idea behind preconditioning is interesting and is supported by animal experimental data.

The most likely application in humans is limb preconditioning before procedures or surgeries that pose threats to perfused organs and tissues. This short-term effect is best supported by animal and human preliminary data.

The application of limb preconditioning to procedures that reduce brain perfusion has not been studied at all using end points that quantify brain perfusion or the frequency and extent of brain infarction.

There are many problems in applying limb ischemic preconditioning to more chronic situations that limit brain perfusion. Preconditioning for 300 days (or even for 100 days or less) is not practical. Furthermore in most patients who have strokes or TIAs, recurrence is most frequent in the few days and weeks after the initial event. The safety of the repeated protracted preconditioning on the limbs has not been studied and may be a problem especially in older stroke patients who are prone to have peripheral atherosclerosis.

## My Suggestions

Acute limb preconditioning can be tested in patients undergoing surgery or procedures that involve the heart, aorta, and neck and intracranial arteries. Studies have shown that a limited number of inductions during a short period of time are safe and can be performed practically. The frequency and extent of brain infarction would be the best and most clinically important end point to study. No human data is now available that can predict the effectiveness of this strategy.

Limb ischemic preconditioning in more chronic situations is much less supported by preliminary data and is much more difficult and impractical to study and carry out. Instead more animal data to better identify the biochemical mediators of the putative neuroprotection is needed before carrying this approach to the clinic.

My guess is that the scheme of using limb preconditioning to prevent strokes will not have a long life but might be very useful in provoking more basic research that better identifies the mechanism of the putative neuroprotective effect. Administering the effective agent or agents induced by the limb ischemia might prove to have much more longevity.
